# Assessing the precision of high-throughput computational and laboratory approaches for the genome-wide identification of protein subcellular localization in bacteria

**DOI:** 10.1186/1471-2164-6-162

**Published:** 2005-11-17

**Authors:** Sébastien Rey, Jennifer L Gardy, Fiona SL Brinkman

**Affiliations:** 1 Department of Molecular Biology and Biochemistry, Simon Fraser University, Burnaby, BC, Canada V5A 1S6

## Abstract

**Background:**

Identification of a bacterial protein's subcellular localization (SCL) is important for genome annotation, function prediction and drug or vaccine target identification. Subcellular fractionation techniques combined with recent proteomics technology permits the identification of large numbers of proteins from distinct bacterial compartments. However, the fractionation of a complex structure like the cell into several subcellular compartments is not a trivial task. Contamination from other compartments may occur, and some proteins may reside in multiple localizations. New computational methods have been reported over the past few years that now permit much more accurate, genome-wide analysis of the SCL of protein sequences deduced from genomes. There is a need to compare such computational methods with laboratory proteomics approaches to identify the most effective current approach for genome-wide localization characterization and annotation.

**Results:**

In this study, ten subcellular proteome analyses of bacterial compartments were reviewed. PSORTb version 2.0 was used to computationally predict the localization of proteins reported in these publications, and these computational predictions were then compared to the localizations determined by the proteomics study. By using a combined approach, we were able to identify a number of contaminants and proteins with dual localizations, and were able to more accurately identify membrane subproteomes. Our results allowed us to estimate the precision level of laboratory subproteome studies and we show here that, on average, recent high-precision computational methods such as PSORTb now have a lower error rate than laboratory methods.

**Conclusion:**

We have performed the first focused comparison of genome-wide proteomic and computational methods for subcellular localization identification, and show that computational methods have now attained a level of precision that is exceeding that of high-throughput laboratory approaches. We note that analysis of all cellular fractions collectively is required to effectively provide localization information from laboratory studies, and we propose an overall approach to genome-wide subcellular localization characterization that capitalizes on the complementary nature of current laboratory and computational methods.

## Background

The identification of a bacterial protein's subcellular localization (SCL) represents an important step in many analyses. Such information may provide clues regarding the function of a protein. It can assist in the design of laboratory experiments to study a particular protein, and in the case of surface-exposed and secreted proteins, it can aid in the identification of potential vaccine candidates, diagnostic agents or antimicrobial targets [1-3]. The rapid and accurate assignment of SCL for a given protein deduced from a genome sequence thus represents an important step in processes ranging from genome annotation to drug discovery.

Several types of laboratory methods are frequently used to identify a protein's localization. Techniques such as immunofluorescence and immunoelectron microscopy [4], PhoA protein fusions [5], fluorescent-protein tagging [6], and the Western/SDS-PAGE [7] analysis of subcellular fractions are often applied to the analysis of either single proteins or a small sets of proteins. While such methods can provide high-quality localization information, they can be costly and/or time-consuming, and the number of proteins for which an SCL can be assigned is relatively low. Recently, proteomics technologies have been developed which are capable of providing SCL information for a much larger number of proteins. Techniques such as two-dimensional gel electrophoresis and mass spectrometry [8-12] have been frequently used to analyze localization for a variety of bacterial genomes, including *Pseudomonas aeruginosa *[13] and *Bacillus sp. *[14]. Many of these studies have focused on distinct cellular compartments, through the analysis of samples obtained by subcellular fractionation ("subcellular proteomics") [15-19]. A major disadvantage of subproteome analyses is that the fractionation of a complex structure like the cell into several subcellular compartments is not a trivial task. Contamination from other cellular compartments may occur and some proteins are known to span multiple localization sites [7,20-25]. Despite these limitations, however, genome-scale techniques are rapid, cost-effective, and capable of returning results for hundreds or even thousands of proteins in a single analysis.

Computational methods have also been developed to aid analysis of protein SCL. While some subproteomic studies have used methods like GRAVY [26], SignalP [27], and TMHMM [28] as a complement to their laboratory results [15,17], these programs predict protein features rather than localization sites, and thus are often of limited utility when attempting to confirm a protein's SCL. Prior to 2003, the only localization prediction method available for bacteria that was capable of assigning a protein to one of several different localization sites was PSORT I [29]. Developed in 1991, the program had not undergone any significant updates since its release, and has a measured precision level of only 59% [30]. To meet the need for a comprehensive, updated and precise bacterial localization prediction tool, we therefore developed PSORTb v.1.0 in 2003 [30], releasing an updated version 2 of the program in 2004 [31]. PSORTb uses a series of 10 Gram-positive and 12 Gram-negative analytical modules to examine a query protein. Each module scans the protein for the presence or absence of a particular feature characteristic of a specific localization site, returning as output either a predicted localization site or – if the feature is not detected – a result of "unknown". The modules include: SCL-BLAST for homology-based detection, the HMMTOP transmembrane helix prediction tool, a signal peptide prediction tool, a frequent subsequence-based support vector machine, as well as motif and profile-matching modules. The predicted localization sites outputted by each module are then integrated by a Bayesian network into a final prediction. The program is able to assign a protein to one of five localization sites in Gram-negative bacteria (cytoplasm, cytoplasmic membrane, periplasm, outer membrane, or extracellular) or to one of four sites in Gram-positive bacteria (cytoplasm, cytoplasmic membrane, cell wall, or extracellular). It is also able to generate predictions of multiple localization sites for a protein that spans two cellular compartments, and if not enough information is available to make a confident prediction, it is able to return a prediction of "unknown". This method was designed to emphasize precision, attaining a measured precision level of 96% for both Gram-negative and Gram-positive bacteria, with a recall of 82%. A database of predictions based on all currently available complete genomes is available through PSORTdb [32]. Subsequently, other methods have been developed for computational prediction of bacterial subcellular localization (see psort.org for a list), including methods with comparable accuracy such as Proteome Analyst [33], though PSORTb remains the most precise method to date. The existence of these new computational methods now requires an evaluation of how well laboratory and computational methods identify proteins of different SCL. For genome-wide analysis, do laboratory and computational methods behave equally or are particular localizations better predicted by one or both approaches? How can we best characterize SCL using these methods for future genome-wide studies?

We therefore compared selected bacterial subproteomic studies with PSORTb-based computational SCL predictions. Our study indicates that high-precision computational methods like PSORTb are now exceeding the precision levels associated with high-throughput 2D gel-based laboratory methods for localization identification. We also observe that there is, however, a useful complementary relationship between the laboratory-based and computational methods, with certain localizations being more accurately identified by one method over the other. Our work also illustrates the importance of examining all localizations in concert, preferably using a combination of both methods, to gain a more accurate view of a given protein's localization in the cell.

## Results

### Comparison of computational and subproteomic-based predictions of SCL for 405 proteins

When computational SCL predictions by PSORTb v.2.0 were compared to the selected subproteomic studies from Gram-negative bacteria (listed in Table 1), 405 proteins were identified which met our selection criteria – the results of the analyses could be matched to specific GenBank records from the organism being studied (see Methods for details). A matrix showing the predicted localization sites for the ten studies is presented in Table 1, together with estimated % agreement and % coverage for each study.

**Table 1:** PSORTb v.2.0 predicted localization sites for 405 proteins reported in ten subproteome studies.

Laboratory Data			PSORTb v.2.0 Predicted Localization^a)^												
Organism	Fraction^a)^	Total	C	C/CM	C/P	CM	CM/P	P	P/OM	OM	OM/EC	EC	UN	Agreement^b)^	Coverage^c)^
*E. coli *[64]	C	23	19	-	-	-	-	1	-	-	-	-	3	95.0	87.0
*Synechocystis *[15]	CM	63	13	2	-	5	1	6	-	5	1	-	30	24.2	52.4
*Synechocystis *[46]	P	57	2	-	1	-	-	8	-	3	-	-	43	64.3	24.6
*K. pneumoniae *[16]	OM	3	-	-	-	-	-	-	-	3	-	-	-	100.0	100.0
*S. typhimurium *[16]	OM	11	2	-	-	-	-	-	-	6	1	-	2	77.8	81.8
*E. coli *[17]	OM	39	3	-	-	-	1	3	-	22	1	1	8	74.2	79.5
*P. gingivalis *[18]	OM	6	-	-	-	-	-	-	-	2	-	1	3	66.7	50.0
*P. aeruginosa *[13]	OM	33	4	-	-	1	-	-	1	22	2	1	2	80.6	93.9
*P. aeruginosa *[13]	EC	150	33	-	-	5	1	33	-	9		6	63	6.9	58.0
*H. pylori *[19]	EC	20	3	-	-	-	-	2	-	4	1	1	9	18.2	55.0

a) C = cytoplasmic, CM = cytoplasmic membrane, P = periplasmic, OM = outer membrane, EC = extracellular, and UN = unknown.

b) Percentage of agreement is defined by , where: A represents the number of proteins of the fraction X predicted by PSORTb to be resident at X and X/Y localization sites.

B represents the total number of proteins of the fraction X predicted as not unknown by PSORTb.

c) Percentage of coverage is defined by , where: B represents the total number of proteins of the fraction X predicted as not unknown by PSORTb.

T represents the total number of proteins identified in the fraction X.

Because PSORTb is designed with an emphasis on high precision, the program returns a prediction of "unknown" if not enough information is available to make a confident prediction. 163 of the 405 proteins being compared, or 40.2%, returned a result of unknown and were not considered in the downstream analyses. Of the remaining 242 proteins, the experimentally observed localization site agreed with the computationally predicted localization site in only 104 cases, for a total % agreement of 43.0%. This figure dropped to 25.7% if the unknown proteins were included in the calculation. The figures vary significantly from study to study, with % agreement ranging from a low of 6.9% (4.0% including unknowns) in the largest study to a high of 100% in the smallest study. However, it is clear that among the 405 proteins, there are likely a significant number of false positives and false negatives.

### Identification of potential contaminants

Subcellular fractionation is a widely-used method for isolating the proteins resident at a specific cellular compartment [34]. However, a significant limitation of the technique is the problem of cross-contamination, in which small amounts of proteins from neighbouring compartments contaminate the fraction of interest [7,21-23]. This leads to the inclusion of false positives in the resulting datasets.

With the computational and subproteomic localizations differing for as many as 93.1% of the proteins for a particular analysis, we suspected that certain subproteome studies we analyzed were prone to cross-contamination. The two studies examining the extracellular fraction, in particular, had a % agreement with the computational predications of only 6.9% and 18.2%, therefore we suspected that contamination may have been a particular problem for these studies. This may be due in part to autolysis, a process common to many bacterial species which release cellular proteins into the extracellular milieu [35]. It may also be due to cellular lysis during the centrifugation of the cells [19]. If we exclude the study with 100% agreement, which involves only a small (n = 3) number of proteins, we observe that the study with the most agreement between the two methods involved an analysis of the *E. coli *cytoplasm. The single possible contaminant observed in this *E. coli *cytoplasmic study suggests that the cytoplasm is the easiest compartment to isolate in a subfractionation analysis.

When a number of subproteome studies of Gram-positive bacteria were analyzed, we observed a similar trend. Of the seven studies we examined [14,36-41], the *Corynebacterium glutamicum *[36] and *Mycobacterium leprae *[38] cytoplasmic subproteome experiments displayed the lowest levels of observed/predicted disagreement, at 0% and 8% respectively. However, when two Gram-positive extracellular fractions were analyzed (*Staphylococcus aureus *[41] and *Bacillus sp. *[14]), the % disagreement was measured at 53% and 33% – figures which are significantly lower than those observed for Gram-negative bacteria.

We next proceeded to examine the 138 disagreeing cases on an individual basis to identify the source of potential false positive results. While many false positive results appeared simply to be the result of "leaky" subfractionation, we did observe a number of cases in which a protein resident in the fraction of interest was identified along with its interacting partners from neighbouring cellular compartments. For example, Molloy et al. [17] report the presence of the acriflavine resistance protein A (AcrA) in the outer membrane fraction however, AcrA – which is predicted by PSORTb to be a cytoplasmic membrane protein – is known to be dually localized in both the cytoplasmic membrane and the periplasm [42,43]. AcrA interacts with the outer membrane protein TolC to form an export system, thus we suspect that AcrA was found in the outer membrane fraction due to its tight association with TolC. Another instance of "co-fractionation by association" was observed with the PilJ protein isolated from the *P. aeruginosa *outer membrane fraction. This protein is predicted by PSORTb to be localized to the cytoplasmic membrane and displays significant similarity to the known cytoplasmic membrane protein methyl-accepting chemotaxis protein II from *Salmonella typhimurium *[44]. PilJ is part of the chemosensory systems of *P. aeruginosa *[45], and it was likely co-fractionated through its association with another component of the chemosensory system present in the outer membrane.

We also observed several conflicting cases amongst the results when closely related proteins were examined. 85 of the 405 proteins in the analysis can be grouped into 36 groups of proteins which appear multiple times in the results. These 36 groups consist of: 1) a single protein identified more than once in the studies (e.g. OprE, identified in both the *P. aeruginosa *outer membrane and extracellular fractions [13]); 2) two or more paralogs (e.g. *Synechocystis *CcmK homolog 1 and CcmK homolog 2, both identified in the cytoplasmic membrane fraction [15]); or 3) two or more orthologs (e.g. *Helicobacter pylori *carbonic anhydrase, identified in the extracellular fraction [19], and *Synechocystis *carbonic anhydrase, identified in the periplasmic fraction [46]).

We would expect these groups of closely related proteins to be isolated from the same subcellular fractions, since subcellular localization is highly conserved across diverse taxonomic lineages [47]. However, this is only the case for 18 of the 36 groups, although 33 of the 36 are predicted by PSORTb to reside in the same localization. Fifteen groups contain related proteins isolated from two different fractions. Two groups (the ATP synthase beta chain proteins and the elongation factor family) contain proteins isolated from three fractions, and one group (the GroEL, GroEL2 and GroES chaperonin proteins) was isolated from four different subcellular fractions. These latter three groups illustrate an important trend with respect to contamination – certain abundant, predominantly cytoplasmic, proteins are repeatedly found in the list of potential contaminants, either due to the subfractionation process or their association (even if temporary) with proteins of another localization (for example, the protein folding chaperones). In the majority of these studies, however, they are not noted as potential contaminants/co-purifying proteins.

Our analysis of false positives reveals that the potential for contamination appears to be lowest when the cytoplasm is the subfraction of interest, and highest when the extracellular fraction is analyzed. The data highlights the fact that employing a computational contaminant screening procedure is a valuable addition to a subproteome analysis. It is especially critical for extracellular analyses, as both autolysis and mechanical lysis of cells during subfractionation can release the contents of other cellular compartments into this fraction of interest. The ubiquitous cytoplasmic proteins ATP synthase beta, elongation factors, and the GroEL/ES chaperonins are frequently observed contaminants; however, many of the studies in which these proteins were identified do not address this fact. While these proteins might immediately raise a flag to most proteomics researchers, they are not commonly noted and so may not be appreciated by genomics researchers using SCL data for genome annotation or cell surface drug target identification. Failure to note these proteins as potential contaminants/co-purifying proteins may also have significant consequences for bioinformatics software development. For example, inaccurate subcellular localization assignments could be propagated if the data were used as training data for a machine learning method by researchers unfamiliar with the field.

### An estimation of the precision of subproteome 2D gel analyses

An interesting figure results from the analysis of the 44 proteins that were both isolated in a subproteome study and are present in the ePSORTdb database [32] of proteins of known subcellular localization. In 12 of these 44 cases, the fraction from which these proteins were isolated in the subproteomic studies did not match the previously reported experimentally verified localization. If we view these 44 proteins found in ePSORTdb as "100% precise predictions", we arrive at a "true" potential contamination rate of 27.3%. Nine of these conflicting results were found in the extracellular fraction in the subproteomic experiments and may represent by-products of cellular lysis. The remaining three proteins were isolated from the *E. coli *outer membrane fraction [17], though they were previously shown to be periplasmic proteins. The authors of this subproteome study propose that these proteins were extracted through their association with outer membrane components, rather than improper fractionation technique.

We then carried out a more liberal analysis by investigating the 138 cases where the PSORTb and subproteomic localizations differed. For each of the 138 proteins, we attempted to determine the most probable actual localization site. Localizations for twelve proteins, mentioned above, were found in ePSORTdb. We next looked for a published report of localization in the literature for the remaining 126 proteins. If no published information was available, we then looked for significant (E > 1e-10) similarity to a protein of known localization.

In this fashion, we were able to confirm that the localization predicted by PSORTb was correct in 87 of the 138 proteins. For the remaining 51 proteins, neither published localization information nor similarity to a protein of known localization was observed, and we were unable to determine whether the PSORTb or subproteomic localization site was correct. The results of this analysis are presented in Table 2.

**Table 2:** Estimation of subproteome study error rate.

Organism	Fraction^a)^	Total proteins identified	Disagreements^b)^	Confirmed PSORTb errors^c)^	Confirmed laboratory errors^d)^	% Errors^e)^
*E. coli *[64]	C	23	1	0	0	0.0
*Synechocystis *[15]	CM	63	25	0	4	6.3
*Synechocystis *[46]	P	57	5	0	1	1.8
*K. pneumoniae *[16]	OM	3	0	0	0	0.0
*S. typhimurium *[16]	OM	11	2	0	2	18.2
*E. coli *[17]	OM	39	8	0	6	15.4
*P. gingivalis *[18]	OM	6	1	0	1	16.7
*P. aeruginosa *[13]	OM	33	6	0	3	9.1
*P. aeruginosa *[13]	EC	150	81	2	36	24.0
*H. pylori *[19]	EC	20	9	1	5	25.0

Total		405	138	3	58	14.3

^a) ^C = cytoplasmic, CM = cytoplasmic membrane, P = periplasmic, OM = outer membrane, and EC = extracellular.

^b) ^Disagreement represents the number of proteins of the fraction X predicted by PSORTb not to be resident at X or X/Y localization sites.

^c) ^Confirmed PSORTb error represents the number of disagreeing cases for which the PSORTb predicted localization site was found to be incorrect.

^d) ^Confirmed laboratory error represents the number of disagreeing cases for which the PSORTb predicted localization site was found to be correct.

^e) ^% Errors is calculated as the number of confirmed laboratory errors divided by the total number of proteins identified.

Using this more liberal analysis, we estimated the average error rate of laboratory subproteome experiments to be 14.3%. Estimated error rate values varied considerably between studies, from a low of 0% (*K. pneumoniae *outer membrane analysis, in which only 3 proteins were investigated) to a high of 25.0% (*H. pylori *study of the extracellular fraction). Again, we observed that extracellular studies appeared to have the highest error rates due to the strong potential for contamination discussed earlier. On average, though, the subproteomic analysis error rate for all localizations was significantly higher than the error rate of 4% previously determined for PSORTb [31].

### Reducing information loss: proteins with dual localization sites

A second disadvantage of subcellular fractionation is the associated information loss. Certain proteins have domains in two or more neighbouring cellular compartments, some may cleave into two products, each residing at a different site [48], and others [20] may be found at different localizations over time, or during different environmental conditions [49]. Because subproteome studies typically address a single cellular compartment, it is quite difficult to identify multiply-localized proteins from the results.

Computational methods can help to reduce the information loss associated with subproteome studies. When a disagreement is observed in cases where the computational and subproteomic localization sites are neighbours, it may indicate a dually localized protein. An example found in the present analysis is the ATP synthase AtpG (beta prime subunit). This protein was extracted from the *Synechocystis *cytoplasmic membrane fraction but was predicted as a cytoplasmic protein by PSORTb. Inspection of the literature reveals that AtpG contains domains located in both the cytoplasm and cytoplasmic membrane [50-52].

PSORTb also flags proteins predicted to reside in two compartments. Thirteen of the 405 proteins are predicted to reside at dual localization sites, with the bulk of these predicted as outer membrane/extracellular. This particular combination of localization sites suggest an autotransporter – a protein with a beta-barrel transporter domain and extracellular globular domain that is cleaved and released after translocating through the pore formed by the transporter domain. Indeed, many of the 13 proteins flagged by PSORTb are known autotransporters, including esterase and the *H. pylori *vacuolating cytotoxin.

Although PSORTb can assist in the identification of dually-localized proteins, false negatives are still possible. If the observed site and the single predicted sites are identical, a protein's secondary localization will still go undetected. Though it may not always be feasible, a potential solution to this problem would be to perform 2D gel analyses of all five compartments in one experiment. Not only would this aid in the identification of proteins with multiple localization sites, a comparison of the amounts of protein present in each fraction could be of use when screening for potential contamination.

### Comparison of PSORTb with previously reported contaminant screening procedures

Our results illustrate that it is important to screen the results of a subproteome study for potential errors. However, many groups do not perform such a screen, or employ approaches which are limited in their utility.

The authors of two of the subproteomic studies analyzed here performed basic contaminant screens. In the *Synechocystis *cytoplasmic membrane study [15], the 63 proteins identified were submitted to TMHMM [28]. Seventeen of these proteins were classified as integral membrane proteins based on the presence of one or more helices. The remaining 46 were annotated as peripherally-associated membrane proteins and were then analyzed by SignalP [27]. Proteins with predicted signal peptides were classified as associated to the periplasmic face of the membrane, while those without predicted signal peptides were classified as peripherally associated to the cytoplasmic face.

Using only a single localization predictive method such as TMHMM to identify a feature often results in false positives, particularly in alpha helix detection, where signal peptides are often mistaken for helices. Furthermore, by describing the proteins with no detected helices as peripherally membrane-associated, there is a failure to recognize the fact that these proteins may represent potential contaminants from other fractions. Had PSORTb been used as a screening tool, the authors would have been able to identify 22 potential errors amongst their results with a relatively high degree of confidence.

The authors of the *E. coli *outer membrane study [17] compared the Swiss-Prot localization site for the proteins they identified to the amounts of those proteins detected on the 2D-gel. They reported that, with the exception of the flagellin protein, only proteins annotated as integral outer membrane proteins were detected in significant levels. They posit that the remaining proteins, detected at lower levels, may exhibit a functional association with proteins in the outer membrane. However, this explanation does not account for several potential cytoplasmic or cytoplasmic membrane contaminants, such as the dihydrolipoamide succinyltransferase SucB [53,54], which were also isolated. A screen such as this also has the potential to produce a high number of false negatives – outer membrane proteins present in low quantities which are mistaken for potential contaminants.

While the authors of the two studies mentioned above do not claim that their approaches identify all contaminants, we found that a robust and comprehensive method such as PSORTb outperforms single methods designed to analyze specific features, such as signal peptides or transmembrane helices. This is not surprising, as it has long been recognized that multi-component approaches to prediction achieve the best performance. Though dually localized proteins likely represent only a small fraction of proteins in the cell, they often represent interesting biological cases, including proteins that play pivotal roles in antimicrobial resistance (i.e. efflux proteins [55]), and virulence (i.e. BrkA [56]) and thus should not be overlooked.

### Optimal identification of cytoplasmic membrane proteins requires a combined computational and laboratory approach

Examining the detailed PSORTb results for the proteins reviewed in the present analysis, we observed an interesting trend in the output of the HMMTOP module, which predicts the number of transmembrane alpha-helices in a query protein. Of the 405 proteins analyzed by HMMTOP, only six proteins contained three or more predicted helices. Even more surprising was that only three of these six were identified in the *Synechocystis *cytoplasmic membrane study. When three cytoplasmic membrane subproteome studies in Gram-positive bacteria were analyzed, the same trend was observed, with only six out of 269, or 2.2%, of proteins predicted to contain three or more transmembrane helices (TMHs). We then analyzed the complete *Synechocystis *proteome with PSORTb, predicting a total of 540 cytoplasmic membrane proteins, of which 461 contain three or more transmembrane helices.

Our results indicate that 2D gel electrophoresis of the cytoplasmic membrane fraction is only capable of identifying a small proportion of the multi-pass membrane proteins in a given proteome, likely due to the low pI and poor solubility of these proteins [57]. While other techniques can be used to identify these proteins in the laboratory – for example, liquid chromatography coupled with tandem mass spectrometry and affinity labelling [58,59] – PSORTb is a cheaper and faster solution which is capable of identifying these proteins with a high degree of precision.

While PSORTb appears to outperform laboratory subproteomic methods for the identification of proteins with three or more transmembrane helices, the opposite is true for membrane-associated proteins with one or two helices. In their analysis of the *Synechocystis *cytoplasmic membrane fraction, the authors of the study report 40 membrane-associated proteins. PSORTb, on the other hand, only confidently identifies three such dually localized proteins – two with cytoplasmic domains, and one with a periplasmic domain. In order to maintain a high level of precision, PSORTb requires that one of the following criteria be met to identify a cytoplasmic membrane protein: three or more predicted TMHs, similarity to a known membrane protein, or a positive result from the cytoplasmic membrane SVM module. As a result of these stringent criteria, a large number of cytoplasmic membrane-associated proteins with one or two helices are not identified by PSORTb.

Our observations indicate that the cytoplasmic membrane presents a special case for both laboratory and computational analysis. If a true picture of the membrane proteome is desired, it is necessary to use a combined approach, in which a computational method is used to identify integral cytoplasmic membrane proteins, while a laboratory method is used to identify cytoplasmic membrane-associated proteins.

## Discussion

### Comparing the precision of laboratory and computational methods

In the present analysis, we compared the localizations predicted by the computational method PSORTb to the localizations of 405 proteins reported in ten subproteome 2D gel electrophoresis studies. The data generated in our analysis indicates that subproteome studies vary greatly in terms of their precision. Certain small studies of particular fractions, such as the analysis of three *K. pneumoniae *outer membrane proteins or 23 *E. coli *cytoplasmic proteins, display low or non-existent apparent error rates. Larger studies and those focusing on particular localizations – including the extracellular milieu – can contain significant levels of false positive, or contaminant proteins.

We attempted to estimate the precision associated with subproteome studies using two approaches. In the first, more stringent approach, a comparison of 44 proteins against the ePSORTdb database of proteins of experimentally verified localization yielded a rough estimate of false positives of 27.3%. A second approach, in which we attempted to determine the true localization of 138 proteins using literature and homology-based approaches, yielded an estimate of 14.3%.

While our approximate error rate is by no means a definitive estimate and was not calculated using large samples, it does illustrate the importance of evaluating the results of a subproteome study with a critical eye. While errors associated with each study do vary, on average as many as 1 out of every 4–7 results could be erroneous.

Even more notable is the observation that while our estimated precision of subproteome analysis exceeds that of early predictive tools such as PSORT I [29] (with a reported precision of 59.6% [30]), current high-precision computational methods such as PSORTb (with 96% precision) appear to outperform laboratory subproteome studies, generating fewer false positive results. While it is true that measured precision values calculated from cross-validation studies of test datasets represent a slight overestimation of precision, even a more conservative estimate of 90% precision still exceeds the levels attained by most high-throughput laboratory methods. In other words, PSORTb, first released in 2003, appears to be the first computational method developed that outperforms high-throughput laboratory studies for SCL prediction. Other computational methods have since been developed that also have high accuracy, and slightly more recall (sensitivity) such as Proteome Analyst. However, no method has yet been developed that is as precise as PSORTb.

### Limitations of computational methods

While our comparison of the precision achieved by computational and laboratory subproteome analyses indicates that certain predictive tools have surpassed wet-bench methods for localization identification, there are a number of caveats associated with the use of computational tools.

Of the 405 proteins submitted to PSORTb, only 59.8% returned a predicted localization site and in only 43% of these cases did the predicted site match the observed site. The 40.2% "unknown" rate we observed is well below the recall of 82% reported in the paper describing PSORTb. Such a discrepancy between "practical" values and "theoretical" values is frequently observed with machine learning methods, due to the fact that the data used to train and test the method is generally quite well-annotated while "real world" data, on the other hand, contains large numbers of hypothetical proteins.

Unfortunately, until machine learning methods – including PSORTb – are trained on much larger datasets, the gap between recall values is not likely to improve significantly. In the interim, we recommend that users employ additional predictive strategies with higher recall values. Proteome Analyst [33] uses a different approach to PSORTb in generating its predictions – keywords are extracted from Swiss-Prot annotations of proteins homologous to a given query; these keywords are then passed to a machine learning classifier. Proteome Analyst displays excellent precision – the authors report an overall precision of 95.9% for Gram-negative bacteria – and although its coverage when applied to whole genomes is generally comparable to PSORTb, it did provide a much larger number of predictions for the dataset analyzed here – of the 405 proteins submitted, Proteome Analyst returned a predicted localization site or sites for 398.

The performance of a given method can also vary significantly depending on the organism being analyzed. For example, PSORTb was able to generate predictions for only 25% of the proteins identified in the *Synechocystis *periplasmic fraction (see Table 1). Several factors may explain this low rate of coverage, including particularities of the morphology of *Synechocystis sp.*, the low number of *Synechocystis *proteins included in PSORTb's training dataset, and the fact that three-quarters of the proteins found in the periplasmic fraction are annotated as hypothetical proteins. This is in contrast to the excellent coverage achieved by PSORTb in the analysis of the *E. coli *cytoplasmic fraction, which reflects the fact that as a model organism, *E. coli *proteins occur frequently in PSORTb's training data.

A method's performance also varies between localization sites and, in general, correlates with the amount of training data available for a given localization. PSORTb performs very well when identifying both cytoplasmic and outer membrane proteins, but is not able to make as many predictions for periplasmic and extracellular proteins. Proteins resident at specific localization sites – for example, the periplasm and the extracellular space – can be similar to the point that differentiating the two based on sequence alone can be difficult.

It is also important to note that every predictive method will generate a certain number of false positive results, and that it is critical to keep the measured precision of a given method in mind when carrying out a computational analysis. For example, some computational methods, such as CELLO [60], have a measured precision of only 71.5% [31].

### Limitations of laboratory methods

Laboratory analyses also carry with them a number of caveats. We have already shown that one of the major disadvantages of subproteomic studies is the potential for contamination via leaky fractionation or lysis. Growth conditions can also affect the results of a subproteome study. Different growth conditions can alter the expression of a particular protein, thus while a subproteome study can provide valuable data about expression under a given condition, they may not yield a global picture of the proteins expressed by a bacterium. The parameters of the experiment can also play a key role in determining which proteins are identified from a gel.

It is critical to choose an appropriate pH gradient for maximum resolution of total proteins, and even then standard methods may not detect or separate low abundance or hydrophobic proteins. Protein complexes can also be problematic if their subunits are difficult to disassociate [57,61,62].

### Proposed method for the optimal characterization of cellular compartments

In the present study, we have shown that computational and laboratory-based analyses of specific cellular compartments complement each other, with each method contributing to improve the accuracy of the other. Although both methods do display certain limitations, each offers a number of significant advantages, which we have summarized in Table 3. In order to capitalize on these advantages, we propose that genome-scale studies aimed at cataloguing the proteins of a particular cellular compartment adopt a complementary approach in which both methods are used.

**Table 3:** Advantages and disadvantages of computational and subproteomic approaches to localization analysis.

**Computational methods**	**Proteomics analysis**
**Advantages**	

Rapid predictions for all proteins deduced to be encoded in a given sequence	Can be performed under different conditions and provide condition-specific information
Detailed information about specific features of proteins, e.g. signal peptides, TMHs	Confirms expression of hypothetical proteins
Identification of potential contaminants in subproteome analyses	Large-scale source of data on SCL for hypothetical proteins that cannot be easily predicted computationally
Identification of hydrophobic integral membrane proteins	

**Disadvantages**	

Does not perform as well (less predictions) when analyzing an organism that is not similar to well studied/model organisms.	Time-consuming
May miss flagging some multiply-localized proteins	Low abundance and hydrophobic proteins not readily detected
Poorly predicts particular localizations for which there is little training data, or the proteins are computationally difficult to differentiate between localizations.	Difficult to accurately identify all proteins found on the gel
Cannot identify condition-specific data on SCL, particularly proteins that change SCL depending on the condition.	One subcellular fraction at once analyzed
	Subfractionation often results in contamination
	Cannot identify multiply localized proteins

With respect to the subproteomic aspect of such a study, we suggest that rather than analyze a single cellular compartment, a study ought to analyze all available compartments. By determining the relative abundance of a protein in each compartment, a researcher will able to quickly flag potential contaminants and identify proteins with complex localization profiles – dual localizations or localization that varies temporally.

After retrieving the set of protein sequences corresponding to the spots on a 2D gel, the proteins should be submitted to a high-precision localization prediction method for analysis. PSORTb is the most precise localization prediction tool available, and its consensus approach allows the user to acquire detailed information about protein features, such as homology to protein of known localization, or the presence of a signal peptide, transmembrane helices, or specific sequence motifs and patterns. Proteome Analyst is a second high-precision method which complements PSORTb well, through the use of an annotation-based approach.

The computationally predicted and experimentally observed localization sites should then be compared. In cases where the computational and laboratory methods disagree, detailed analysis of the individual protein should be carried out. Through examination of the literature and further computational analysis, very often a confident call regarding the protein's true localization can be made. An excellent model is provided by Elias et al. [63], who employ a multi-faceted approach – including PSORT I, PSORTb, and in-depth examination of individual proteins – to the analysis of their results from a study of *Shewanella oneidensis *hypothetical proteins.

The combination of 2D gel analysis and PSORTb prediction can provide a remarkably clear and genome-scale picture of protein localization in a given bacterium. Of course, these methods are no replacement for the hypothesis-driven detailed investigation of individual proteins. Instead, they provide an accurate jumping-off point for the in-depth analysis of specific proteins using additional techniques. As both computational and laboratory high-throughput approaches improve in terms of both precision and recall, however, we see an increasingly important role for these methods in the fields of molecular biology and genomics.

## Conclusion

We have performed the first focused comparison of genome-wide laboratory/proteomic and computational methods for subcellular localization identification, and show that PSORTb is the first computational method to attain a level of precision exceeding that of high-throughput laboratory approaches. We note that analysis of all cellular fractions collectively is required to effectively provide localization information from laboratory studies, and we propose an overall approach to genome-wide subcellular localization characterization that capitalizes on the complementary nature of current laboratory and computational methods.

## Methods

### Selection of subproteomic studies

Eight manuscripts describing the 2D gel electrophoresis analysis of ten bacterial subcellular fractions were selected for the present study (Table 1). The studies were chosen to ensure that they represented all five of the possible Gram-negative localization sites over a range of organisms, including: *Escherichia coli*, *Helicobacter pylori*, *Klebsiella pneumoniae*, *Porphyromonas gingivalis*, *Pseudomonas aeruginosa*, *Salmonella typhimurium*, and *Synechocystis*. In all, eight studies were selected [13,15,16,18,19,46,64] spanning all five localization sites for Gram-negative bacteria. In addition, seven supplementary Gram-positive studies were evaluated to a lesser degree to ensure that the results were generally applicable to all bacteria. A total of 269 proteins from the cytoplasm of *C. glutamicum *[36,37] and *M. leprae *[38], from the cytoplasmic membrane of *Bacillus anthracis *[39], *M. leprae *[38] and *Mycobacterium tuberculosis *[40], and from the extracellular fraction of *Bacillus sp. *[14] and *S. aureus *[41], were analyzed. The vast majority of the studies used fractionation followed by two-dimensional SDS-PAGE electrophoresis. Proteins were then subjected to peptide mass fingerprinting (PMF) identification. One study [18] used fractionation followed by two successive one-dimensional SDS-PAGE electrophoresis analyses, with subsequent N-terminal amino acid sequence analysis.

### Protein selection

For each study, we examined the reported proteins to see if they met two criteria. First, the protein must have been identified through comparison of the spot to the sequence of the bacterial genome under study, and not to another organism. For example, in the *S. typhimurium *outer membrane study of Molloy et al. [16], only the proteins identified by a PMF search against the *S. typhimurium *genome were selected, while proteins identified by a PMF search against other organisms were not included. Second, we had to be able to match the protein reported in the study to a GenBank record in order to retrieve the correct amino acid sequence. After these two filtering steps were applied, the final dataset consisted of 405 proteins for the Gram-negative organisms.

### Computational analysis

Computational predictions of localization were performed using the standalone version of PSORTb v.2.0 [31]. The complete predictions are available as supplemental material (See Additional file 1: PSORTb complete predictions). Proteins predicted to reside at multiple localization sites were manually identified from the PSORTb results. A protein was annotated with dual localizations if PSORTb returned two sites with scores between 4.50 and 7.49 or if the SCL-BLAST module returned significant similarity to a protein known to have dual localizations. Additional limited computational analyses were performed with Proteome Analyst [33], as described in the text.

## Authors' contributions

SR selected the subproteomic studies, carried out the computational predictions and performed their analyses. SR and JLG drafted the manuscript and JLG supplied additional insights regarding the analyses. FSLB coordinated the study and provided further insights when refining the manuscript. All authors read and approved the final manuscript.

## Supplementary Material

Additional file 1 **PSORTb complete predictions **The Excel spreadsheet contains the PSORTb v.2.0 detailed predictions for the 405 proteins reviewed in this study. Click here for file
